# Inoculum source dependent effects of ericoid, mycorrhizal fungi on flowering and reproductive success in highbush blueberry (*Vaccinium corymbosum*)

**DOI:** 10.1371/journal.pone.0284631

**Published:** 2023-04-19

**Authors:** Erin O’Neill, Alison K. Brody, Taylor Ricketts

**Affiliations:** 1 Biology Department, University of Vermont, Burlington, VT, United States of America; 2 Gund Institute for Environment, Rubenstein School of Environment and Natural Resources, University of Vermont, Burlington, VT, United States of America; National Institute of Agricultural Research - INRA, MOROCCO

## Abstract

Most terrestrial angiosperms form mutualisms with both mycorrhizal fungi and animal pollinators. Yet, the effects of mycorrhizae on pollinator behavior and plant reproduction are unknown for most species, and whether the source or type of mycorrhizal fungi affects reproductive success has rarely been examined. We examined whether inoculating highbush blueberry (*Vaccinium corymbosum*; Ericaceae) with ericoid mycorrhizal fungi enhanced investment in flowering and attractiveness to pollinators, and thus reduced their levels of pollen limitation over that of non-inoculated plants. We also examined the degree to which pollen limitation was dependent on inoculation source and the surrounding pollinator community context. Three-year-old saplings of *Vaccinium corymbosum* ‘Bluecrop’ or highbush blueberry (Ericaceae) were inoculated with a) ericoid mycorrhizal fungi within soil of the rhizosphere of plants growing at a local blueberry farm, b) a commercially available ericoid inoculant, c) both the local soils and commercial inoculum, or d) were not inoculated and served as controls. They were grown for one year in pots in a common garden and, in the following year, were moved to six farms in central Vermont that were known from prior studies to differ in pollinator abundance and diversity. We conducted a hand pollination experiment at each farm to examine if inoculation or pollinator abundance (i.e., farm context) affected reproductive success. Plants treated with all types of inoculums were more likely to flower, and produced more inflorescence buds than non-inoculated plants in 2018. However, in 2019, plants in the combination inoculum treatment, alone, produced more inflorescence buds than those in the other treatments. Neither the source of inoculum nor hand pollination affected fruit set (the proportion of flowers setting fruit), or fruit sugar content. Hand pollination, but not inoculation, increased berry mass and the average number of seeds produced/berry. Our results add to the growing body of evidence that mycorrhizal fungi can affect reproductive traits of their hosts but that the effects of mycorrhizal fungi depend on the mycorrhizal symbionts.

## Introduction

Most plants interact with multiple other organisms and do so simultaneously. Among the most ubiquitous of multi-species interactions are those of plants, pollinators, and mycorrhizal fungi. Over 85% of all terrestrial flowering plants are animal-pollinated [1] and nearly as many (> 80%) engage in intimate symbioses with mycorrhizal fungi [2, 3].

The interactions with both pollinators and mycorrhizal fungi are critical for plant success. Association with mycorrhizal fungi allows plants to obtain otherwise inaccessible nutrients [4], provide protection from herbivory and disease in some cases [5–7] and, can enhance plant growth [8–10]. Likewise, interactions with pollinators are critical to plant ecology and evolution [1] and have been a driving force in the diversification of angiosperms [11]. Despite the profound effects of both mycorrhizae and pollinators on their hosts, the ways in which these below and aboveground mutualists interact remain an area of relatively new interest and one for which there is much to discover [12–15].

There are a variety of mechanisms by which mycorrhizae could enhance floral traits and affect plant reproduction [15]. Access to greater resources could enhance floral display, and the production of signals and rewards to pollinators, increase nectar production by mitigating drought stress, and allow plants to invest more in fruits and seeds (reviewed in [15]). Although in some cases, mycorrhizae enhance pollinator rewards [16–18], these effects have not been found in other cases [19]. Furthermore, mycorrhizae may alter interactions with floral visitors in species-specific ways with some species responding strongly to the symbioses (or removal thereof) and others responding little or not at all [12, 13, 20, 21].

The ecological and evolutionary outcome of mycorrhizae may also depend on the match between the fungus and its host. Several experiments with potted plants demonstrated that plants perform better when inoculated with soil from their native range rather than inocula from other habitats or a commercial inoculum [22–25]. Each inoculum is likely to contain unique fungal taxa, which vary in their uptake of essential plant nutrients and thus their effects on host plant functional traits. For example, when treated with native inoculum, *Allium cernuum* plants had more leaves and, *Lespedeza capitata*, had greater mean shoot length when compared to plants treated with a commercial inoculum [25]. Similarly, *Aster laevis* plants inoculated with native fungi, had significantly higher dry weights compared to control plants in phosphorous limited environments, suggesting that native fungi compensated for low levels of soil phosphorous [23].

Linking the source or identity of mycorrhizal fungi to functional traits that affect floral visitors has been done in only a few studies and with varied results. For example, inoculation with commercial mycorrhizal fungi increased flower production in *Medicago truncatula* [26] and *Antirhinum majus* [27]. Inoculation at planting also increased flower production in *Vaccinium corymbosum* but only for some host cultivars [28]. Neither local nor commercial inoculum increased flower production in *Salvia columbariae* [29], and native mycorrhizal inoculum *decreased* flower production in *Cucumis sativus* in comparison to the commercial inoculum [30]. In the few studies linking inoculation to successful pollination, inoculation increased the percentage of flowers that set fruit in some [31] but not all cases [18]. Post-pollination, a positive association between mycorrhizae and pollination success may be directly related to mycorrhizae enhancing plant resource status and investment in fruits [32, 33].

Furthermore, the outcome of the interactions between mycorrhizae and pollinators is likely to be affected by the communities and ecosystems in which they occur [13]. The diversity and abundance of pollinator communities depends on the composition of surrounding land-cover, landscape complexity, and habitat fragmentation [34–37]. These landscape characteristics affect the distribution and abundance of floral resources and nesting substrates, both of which are critical in supporting and structuring bee communities. The pollinator community, along with the degree to which plants are inherently pollinator-limited, may affect the links among mycorrhizae, floral traits, and plant reproductive success.

*Vaccinium corymbosum* (Ericaceae), commonly known as highbush blueberry, associates with ericoid mycorrhizal fungi [38] and is pollinated by social and solitary bees [39]. Inoculation of *V*. *corymbosum* with ericoid mycorrhizal fungi affects the way in which plants utilize soil nutrients [40], and enhances floral display size, fruit production, and fruit size, but in cultivar-specific ways [28]. Building on these prior results, we asked whether inoculation-induced enhancement of floral displays altered subsequent interactions with pollinators and affected resulting reproductive success. In addition, we asked whether these effects depended on the ecological context of pollinator abundance and diversity. Last, we asked if the responses of plants to inoculation depended on the type of inoculum used. We hypothesized that inoculation with ericoid mycorrhizal fungi enhances the reproductive success of highbush blueberry (*Vaccinium corymbosum*). We further hypothesized that fungi from native soils, taken from the rhizosphere of *V*. *corymbosum*, provide greater benefit than commercial inoculum.

## Materials and methods

To examine if inoculation of *Vaccinium corymbosum* with ericoid mycorrhizal fungi enhanced bud, flower, and fruit production, we conducted the following experiments. In March of 2018, 360, 3-year-old *V*. *corymbosum* cv. Bluecrop plants were obtained from Hartmann’s Plant Company, Lacota, Michigan, USA. Plants were randomly assigned to one of four treatments: 1) inoculated with commercial ericoid inoculum (Plant Health™) which includes spores of *Hymenoscyphus ericae* and *Oidiodendrum griseum* (N = 90), 2) inoculated with soil from a local farm taken from the rhizosphere of *V*. *corymbosum* cv. Bluecrop plants (N = 90), 3) a combination of the first two treatments (N = 90), 4) a non-inoculated control (N = 90). Plants were removed from their pots, the soil washed from the roots, and the remaining root ball covered with ca. 6 oz of inoculum, soil, or peat, which was applied by hand to the wet roots before placing them in a 7-gallon pot filled with a customized potting mix that was 12:6:3:1 peat:compost:perlite:vermiculite. Plants were then placed outdoor in 10 x 9 arrays and grown for the remainder of the summer. To overwinter, plants in their pots were placed in individual holes in the ground and the pots covered with a combination mulch of spruce, fir, and pine. Plants were fertilized before fruiting each year with 10 mL of SUPERThrive (4:1:1; NPK) fertilizer diluted to field recommended concentrations of 5.5 g/L [40].

Highbush blueberry and other members of the Ericaceae pre-form buds in the fall [41, 42]. Thus, to assess the effects of inoculation treatment on reproduction, the number of overwintering inflorescence buds were enumerated in November 2018 and 2019, as well as the number of flowering inflorescences, and the total number of flowers in both June 2018 and June 2019.

To test the hypothesis that inoculation with ericoids alters reproduction through its effects on pollinators and whether these effects depend on the pollinator community, we combined pollinator observations with hand pollination experiments in 2019 at six different farms located in Central Vermont known to differ in pollinator abundance and diversity [43]. Prior to flowering, 15 plants from each of three treatments (non-inoculated controls, inoculated with commercial ericoid fungi, and inoculated with local soil) were placed at each farm, adjacent to but separate from a field of highbush blueberries.

Plants were observed for 30-minute time blocks, between the hours of 9:00 and 14:00, 3 days per week for the full flowering season from 31 May through 17 June; the time of observation was rotated randomly among treatments and by farm each week. Observations were taken only when conditions were conducive for pollinators—i.e., temperatures were above 10°C and in the absence of rain or high winds. Floral visitors were identified as one of the following: queen *Bombus*, worker *Bombus*, orange *Bombus*, *Megachile*, or *Andrenid* (following [43, 44]). In addition, for each visiting individual we recorded the number of flowers visited and the total time spent visiting each plant.

In some years, farms differed in pollinator abundance and in pollen limitation [43]. Therefore, hand pollination experiments were conducted to examine the degree of pollen limitation as a measure of the importance of inoculation on context-dependent pollination. Branches of each plant were assigned to one of two treatment groups: “hand pollination” in which pollen was artificially added to stigmas, or “open pollination” in which no pollen was added but the flowers were manipulated in the same way otherwise. The treatments were repeated every 2–3 days. Pollen was gathered using a VegiBee™ sonicator which imitates the vibration of bees’ thoracic muscles and elicits the releases of pollen. Pollen was collected onto petri dishes from a variety of plants at each farm to imitate natural bee foraging behavior and was not collected from experimental potted plants. Pollen was then applied to stigmas of flowers using a thin, artist’s paintbrush.

At the end of the season, all berries collected were scored as ripe, or aborted (if shriveled). Average berry mass, berry sugar content, and fertilized seed number were assessed for five berries/plant in 2018 and five berries/branch in 2019. The number of berries collected represented more than 50% of all berries produced by most plants. Seeds were counted using a dissecting scope and scored as small, unfertilized ovules, or fertilized, fully formed seeds (larger, darker colored seeds; [45]).

### Statistical analyses

All statistical analyses were conducted in R 4.2.1 [46]. Using the “lmerTest” package, the effect of inoculation on plant response variables including total flower production, flower size, fruit number, and the proportion of flowers that set fruit were analyzed using each as a response variable and inoculation treatment as the main effect, along with farm, in separate linear mixed effects models. Percent fruit set data were arcsine square-root transformed to improve normality. A linear mixed effects model was used to test the effects of inoculation and year on total number of inflorescence buds formed using plant number as a random effect.

Berry mass and sugar content were used as response variables in linear mixed effects models with pollination treatment, inoculation treatment, and farm as main effects. The average berry mass and sugar content were calculated for the hand pollination and open pollination treatment branches for each plant before analysis. To examine if inoculation treatment, hand pollination, or farm, affected the number of seeds produced by plants, the average number of seeds/berry was used as the response variable in a Poisson-distributed generalized linear mixed effects model with inoculation treatment, pollination treatment and farm as main effects. Interaction effects were tested and, when found to be insignificant, were removed from the model. Package “emmeans” was used to compare berry traits among individual treatment groups.

To examine if inoculation altered the patterns of visitation, a linear mixed effects model was used with the number of visits per flower and the total number of seconds spent on each plant as response variables, farm and mycorrhizal treatment as fixed main effects, and week as a random effect. Visits per flower and time spent per plant were averaged over each plant for each day of observation.

The importance of inoculation, hand pollination, and pollinator context (farm) on reproduction was examined by using fruit set (the proportion of flowers that produced berries) as a dependent variable in a linear mixed effects model. Inoculation treatment, hand pollination treatment, and farm were included as fixed effects. All two- and three-way interactions were tested but none were significant and thus were removed from the model.

## Results

**Fig 1:**
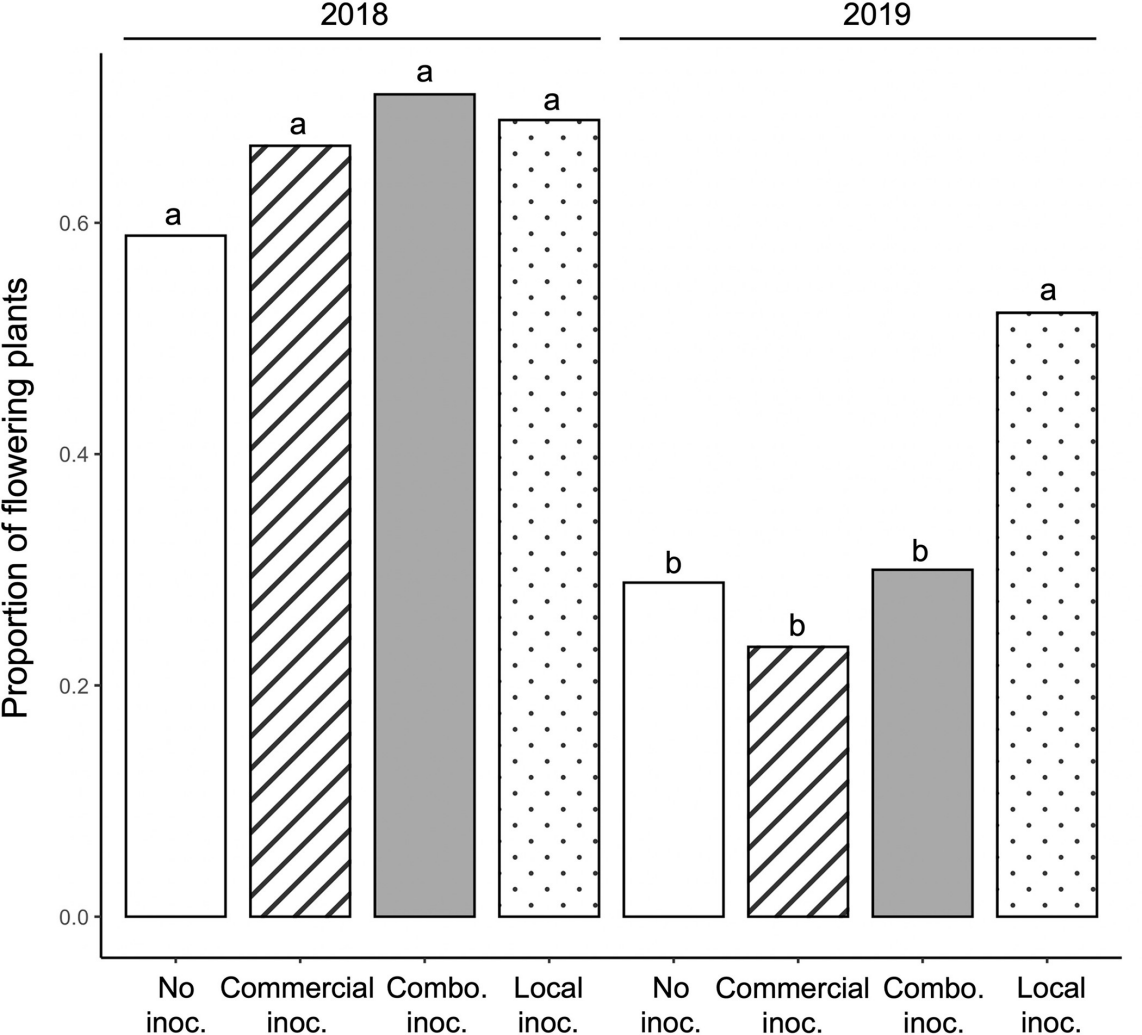
Proportion of plants that flowered in 2018 and 2019 in each of the four treatments. Results were analyzed using an analysis of variance. Letters denote significant differences among treatments and year using a post-hoc Tukey’s HSD test. The combination soil treatment is abbreviated as “Combo inoc.”.

**Fig 2:**
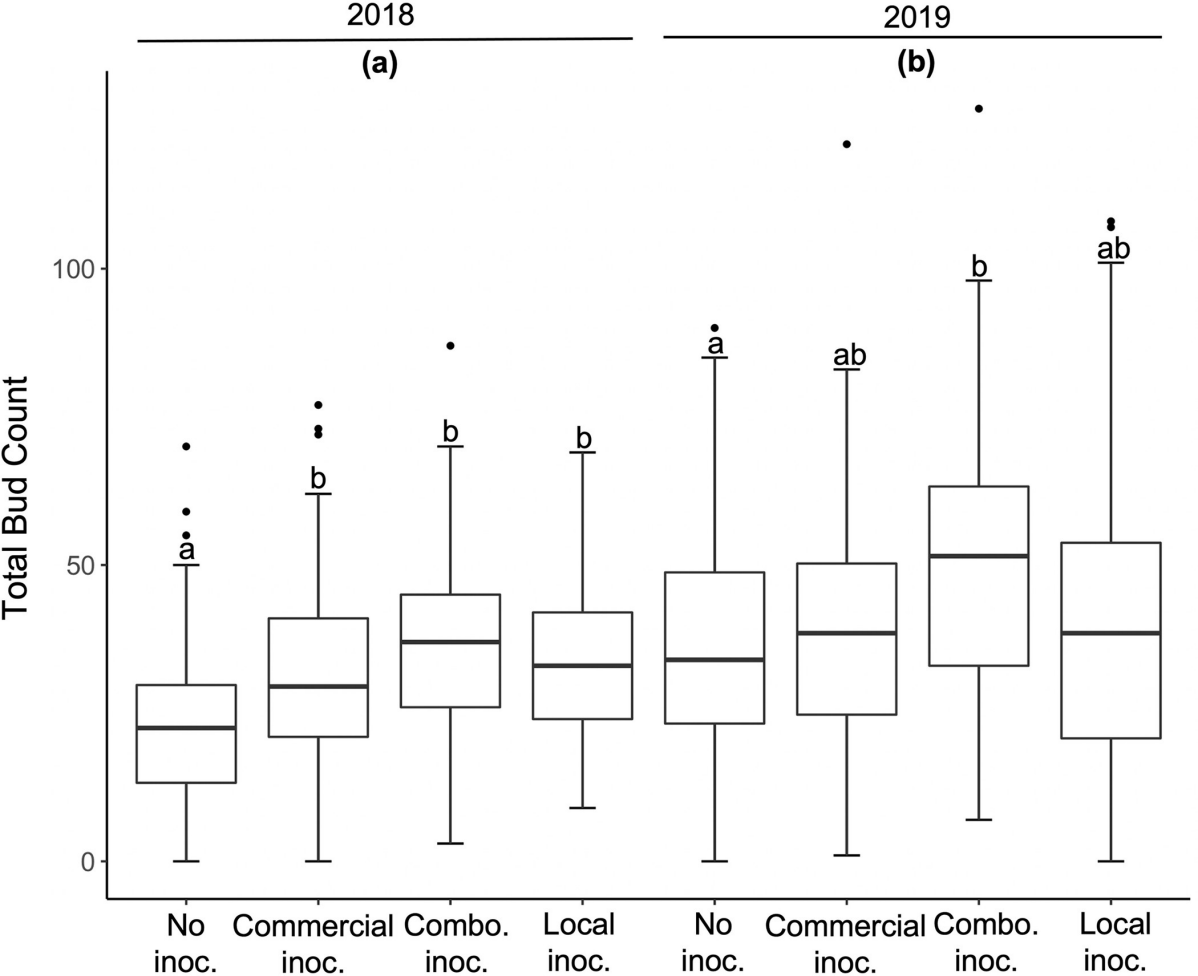
The mean number of inflorescence buds ± 1 standard error formed per plant in each of the four treatments in 2018–2019. Results were analyzed with a linear mixed effects model. Letters denote significant differences among treatments within years groups using R package “emmeans”.

Inoculation with local soil inoculum increased the probability a plant would flower in 2019. Inoculation treatment affected the number of plants that flowered (F_1,720_ = 85.269, P < 0.0001; Fig 1), although the number of plants that flowered differed between years (55.6 in 2018 vs. 31.1% in 2019; F_1,720_ = 85.269, P < 0.0001; Fig 1). The percentage of plants that flowered in 2019 for plants in the local inoculum treatment declined by 15% compared to a 30% decline in those with the commercial inoculum (Fig 1). Inoculation (F_3,589_ = 13.219; P < 0.001; Fig 2) and year (F_1,589_ = 24.459; P < 0.001; Fig 2) also affected the number of inflorescence buds although the effects of inoculum type was inconsistent among years (Fig 2). Add stats on interaction between year and inoc (results comment). Plants inoculated with the combination treatment produced over 25% more buds than the non-inoculated controls in both years (Fig 2), as did those in all the inoculation treatments in 2018 (P < 0.001; Fig 2). On average, plants produced roughly 30% more inflorescence buds in 2019 than in 2018 (P < 0.001; Fig 2) but fewer of these became flowers.

Although inoculation enhanced flowering, neither inoculation treatment (F_2,29_ = 0.736, P = 0.488; S1 Table), nor farm (F_5,29_ = 1.513, P = 0.216; S1 Table), affected the number of pollinator visits per flower a plant received (S1 Table). In addition, inoculation did not affect the amount of time a floral visitor spent on a flower (F_2,29_ = 1.563, P = 0.227; S1 Table), nor did farm (F_2,29_ = 0.766, P = 0.582; S1 Table).

**Fig 3:**
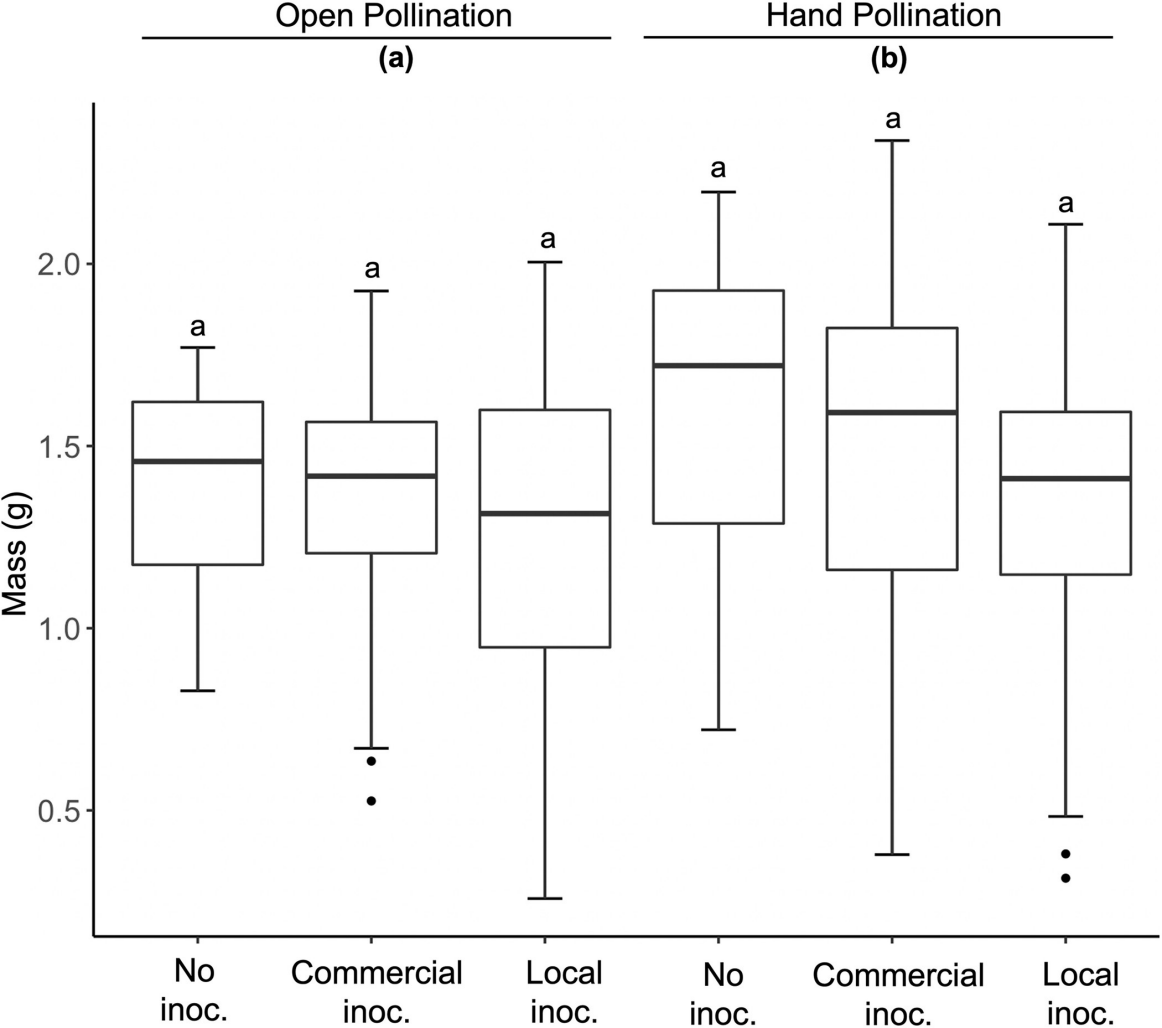
The mean individual berry mass for the non-inoculated control, commercial inoculum, and local soil in 2019. Results were analyzed using a linear mixed effects model. Letters denote differences among treatment groups using R package “emmeans”.

Fruit set was not affected by inoculation treatment (F_5,254_ = 0.1.475, P = 0.23; Table 1), hand pollination (F_2,254_ = 0.784, P = 0.46; Table 1), or by farm (F_2,254_ = 2.08, P = 0.07; Table 1), and none of the interactions between inoculation treatment, hand pollination, or farm, were significant (P > 0.05). Neither inoculation treatment nor farm had significant effects on berry mass (mean = 1.371 ± 0.034; inoc. treatment: F_2,91_ = 0.586; P = 0.558; farm: F_5,84_ = 1.530; P = 0.189; Table 1, Fig 3) or the average sugar content per berry (mean = 11.411 ± 0.157; inoc. treatment: F_2,86_ = 1.312; P = 0.275; farm: F_5,80_ = 0.948; P = 0.455; Table 1). Hand pollination treatment, however, affected both berry mass (F_1,68_ = 7.551; P = 0.007; Table 1, Fig 3) and sugar content (F_1,73_ = 0.033; P = 0.857; Table 1).

**Table 1:** Effects of inoculation treatment (InocTrt), hand pollination treatment (HPTrt), and farm on fruit set, individual berry mass, sugar content, and total seeds per berry.

***Fruit set***	*Df*	*SumSq*	*MeanSq*	*Fvalue*	*Pr(>F)*
*InocTrt*	2	0.017	0.009	1.475	0.231
*HpTrt*	2	0.009	0.005	0.784	0.457
*Farm*	5	0.061	0.012	2.083	0.068
***Mass***	*Df*	*Sum Sq*	*Mean Sq*	*F value*	*Pr(>F)*
*InocTrt*	2	0.066	0.033	0.586	0.558
*HPTrt*	1	0.424	0.424	7.551	0.007
*Farm*	5	0.429	0.086	1.530	0.189
***Brix***	*Df*	*Sum Sq*	*Mean Sq*	*F value*	*Pr(>F)*
*InocTrt*	2	6.091	3.045	1.312	0.275
*HPTrt*	1	0.076	0.076	0.033	0.857
*Farm*	5	10.999	2.199	0.948	0.455
***TotalSeeds***	*Df*	*Chi Sq*		*Pr(>Chisq)*	
*InocTrt*	2	1.101		0.577	
*HPTrt*	1	4.567		0.033 *	
*Farm*	5	2.958		0.706	

**Fig 4:**
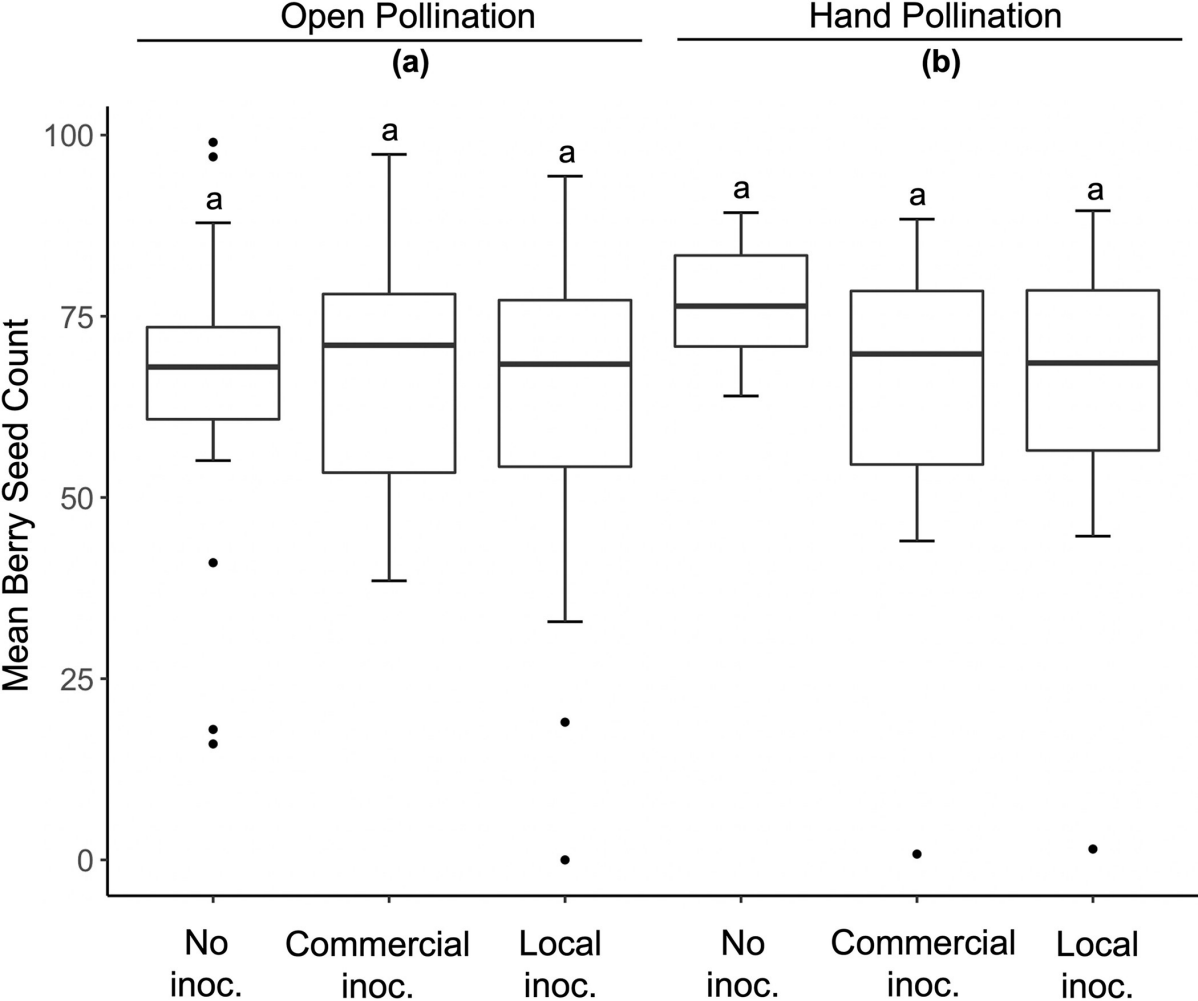
The mean individual berry seed counts for the non-inoculated control, commercial inoculum, and local soil in 2019. Results were analyzed using a Poisson-distributed generalized linear mixed effects model. Letters denote differences among treatment groups using R package “emmeans”.

The average number of seeds/berry had a mean of 66.989 ± 1.409 and was not affected by inoculation treatment (P = 0.577; Table 1, Fig 4) or farm (P = 0.706; Table 1) but was affected by hand pollination (P = 0.033; Table 1, Fig 3). The open pollinated control branches had fewer average seeds per berry (mean = 65.276 ± 2.109; Table 1, Fig 4) than the hand pollinated branches (mean = 68.679 ± 1.865; Table 1, Fig 4).

## Discussion

The presence and identity of mycorrhizal symbionts can affect a suite of traits important to pollinators and plant reproductive success (e.g., reviewed in [15]). In this study, inoculation with ericoid mycorrhizal fungi enhanced plant investment in flowering and altered reproductive success. Plants inoculated with mycorrhizal fungi produced more floral buds and were twice as likely to flower. These effects were dependent, however, on the source of the inoculum and the same inoculum did not produce consistent effects across all responses measured.

Plants responded more strongly to the local soil inoculum and the combined, local soil plus commercial inoculum than to the commercial inoculum alone. In 2019, plants treated with the combination of local soils and commercial inoculum produced more buds than those in the other treatments. In the same year, the number of plants in the local soil inoculum treatment that flowered was almost double that of plants in the other treatments.

These results underscore the variable effects of mycorrhizae demonstrated in other systems as well [16, 18, 22]. Plant response to mycorrhizal fungi can depend on the plant host [16, 18, 47], the fungal symbionts [48, 49], or both [22]. For example, inoculation enhanced investment in flowering either by increasing inflorescence size or the number of flowers in two species of annual plants, while in the third it enhanced the production of nectar compared to those grown without arbuscular mycorrhizal (AM) fungi [16]. In the gynodioecious, *Geranium sylvaticum*, one AM fungal symbiont increased seed number and seed mass in female plants, while the other reduced both fitness parameters in females, hermaphrodites, and the intermediate phenotype [49]. In our study, because we used the same host cultivar, and plants are most often propagated by cuttings, the differences found were likely due to differences in fungi rather than the plants.

Because pollinators often respond positively to floral display [50, 51], the differences among treatments in flowering were expected to translate into higher visitation rates to inoculated plants. However, that was not the case. Although inoculated plants produced more flowers than non-inoculated plants, there were no significant differences among treatments in the number of visits a plant received, the number of flowers visited, or the time pollinators spent per plant. One possibility is that pollinators may respond as much or more to floral rewards as to floral display size [52–54]. In highbush blueberry, per-flower visits were positively correlated with nectar volume [55], although in that study no comparison in flower number per plant was made. For other species, mycorrhizal fungi can positively affect nectar production [16, 21] but we did not measure that variable.

How and whether floral display affects visitation, and the degree to which pollinators limit plant reproduction, may also depend on pollinator diversity and abundance in both natural [56] and managed [43, 44] systems. To examine if differences in the pollinator communities mediated the effects of inoculation on visitation and/or pollen limitation, we intentionally worked at farms where the abundance and diversity of pollinators differed [43, 44]. However, neither inoculation treatment, nor farm, affected any of the response variables measured and hand pollination increased only berry mass and the average number of seeds/fruit.

Fewer plants flowered in 2019 than in 2018. The most likely cause for this was that the plants were infected by two fungal diseases, *Fusicoccum putrefaciens* and *Phomopsis vaccinii*, during the early spring of 2019. It was a cool and wet spring which are ideal conditions for fungal diseases like *F*. *putrefaciens* and *P*. *vaccinii* to spread [57]. These diseases reduced the number of plants that flowered in each treatment after buds had already been pre-formed. The reduction in flowering in the two summers was less for plants growing in the local soil inoculum (17%) compared to those in the combination treatment (24%) and those inoculated with commercial inoculum (34%). Whether the differences in our study arose by different fungal genotypes affecting host defense against disease, we do not know, but arbuscular mycorrhizal fungi have been shown to offer protection from disease [58, 59]. Our results suggest that the local inoculum was protective against the fungal pathogens and/or better adapted to Vermont conditions and, therefore, better mutualists in providing protection to plants.

Our results and those of others [22, 25, 40, 60, 61] suggest that the benefits plants receive may depend on host and/or mycorrhizal fungi genotypes. Highbush blueberry cultivars are known to show a high degree of fungus-host specificity and a high degree of variability in association with ericoid mycorrhizal fungi [40]. In our study, because we used a single plant host, and cultivars of highbush blueberry are often grown from cuttings and thus genotypically the same, the discrimination is likely on the part of the host plant for different fungal genotypes rather than fungi discriminating for or against their host. Although the commercial inoculum is a general inoculum for plants within the Ericaceae and contains fungal spores from species that are ubiquitously associated with *Vaccinium* spp., the local soil inoculum was taken from the rhizosphere of plants of the same cultivar and from an area abiotically similar to where the plants were subsequently grown. Thus, it may be locally adapted to Vermont conditions. The importance of the combination and complementarity of fungi and hosts has been demonstrated in other studies [22, 62–64], overturning prior assumptions that the association between plants and their mycorrhizal symbionts are always mutualistic.

Our results add to the growing evidence that plant interactions with belowground organisms can affect those aboveground. In addition, our results demonstrate that the outcome of the interactions can depend on identity of the fungal partners, the match between fungi and host, and interactions beyond plant host and mycorrhizal fungi such as those with pollinators and plant diseases. The complexity of these interactions challenges generalization and points to the need for greater study at scales that range from molecular mechanisms to functional traits within hosts and fungi, to effects that can only be elucidated in communities and ecosystems.

## Supporting information

S1 Table Mean ± standard error for percent of flowers visited by pollinators and time spent per flower (seconds) for six farms and three treatment groups. No differences were found between groups. Values denoted as “n.e.” were non-estimable due to lack of data.
(DOCX)
