# Supplementary material for: Inoculum source dependent effects of ericoid, mycorrhizal fungi on flowering and reproductive success in highbush blueberry (Vaccinium corymbosum)
Source: PLoS One. 2023 Apr 19;18(4):e0284631. doi: 10.1371/journal.pone.0284631 (PMC10115346; doi:10.1371/journal.pone.0284631)
Supplement: S1 Table — No differences were found between groups. Values denoted as “n.e.” were non-estimable due to lack of data. (DOCX) [file pone.0284631.s001.docx]

**S1 Table.** Mean ± standard error for percent of flowers visited by pollinators and time spent per flower (seconds) for six farms and three treatment groups. No differences were found between groups. Values denoted as “n.e.” were non-estimable due to lack of data.

| **Treatment** | **No Inoculum** | | **Commercial Inoculum** | | **Local Inoculum** | |
| --- | --- | --- | --- | --- | --- | --- |
| **Farm** | % flowers visit | Time spent per flower | % flowers visited | Time spent per flower | % flowers visited | Time spent per flower |
| 1 | n.e. | n.e. | 0.75 ± 0.11 | 3.23±0.73 | 0.16 ± 0.04 | 2.36±0.32 |
| 2 | n.e. | n.e. | n.e. | n.e. | 0.53 ± 0.45 | 2.24±0.51 |
| 3 | 0.11±0.04 | 330.50±329.50 | 0.01 | 1.00±0 | 0.25 ± NA | n.e. |
| 4 | 0.15±0.1 | 9.55±1.79 | 1.07 ± 0.92 | 4.78±1.45 | 0.39 ± NA | 4.73±0.76 |
| 5 | n.e. | n.e. | n.e. | n.e. | n.e. | 5.99±1.72 |
| 6 | 0.32±0.07 | 15.32±2.39 | 0.21 ± 0.12 | 5.16±2.02 | 0.29 ± 0.08 | 32.19±12.81 |
